# Direct and indirect vaccination effects on SARS-CoV-2 infection in day-care centres: Evaluating the policy for early vaccination of day-care staff in Germany, 2021

**DOI:** 10.1017/S0950268823000638

**Published:** 2023-05-04

**Authors:** Anja Schoeps, Jan Walter, Manfred Vogt, Stefan Bent, Philipp Zanger, the Palatina Public Health Study Group

**Affiliations:** 1 Landesuntersuchungsamt Rheinland-Pfalz, Koblenz, Germany; 2 Heidelberger Institut für Global Health, Universitätsmedizin, Heidelberg, Germany; 3 Postgraduate Training for Applied Epidemiology, Department of Infectious Disease Epidemiology, Robert Koch Institute, Berlin, Germany; 4 European Programme for Intervention Epidemiology Training, European Centre for Disease Prevention and Control, Stockholm, Sweden

**Keywords:** SARS-CoV-2, Vaccination, Child Daycare Centers, Staff, Interrupted Time Series Analysis, Transmission

## Abstract

To mitigate the known high transmission risk in day-care facilities for children aged 0–6 years, day-care staff were given priority for SARS-CoV-2 vaccination in Rhineland-Palatinate, Germany, in March 2021. This study assessed direct and indirect effects of early vaccination of day-care staff on SARS-CoV-2 transmission in daycares with the aim to provide a basis for the prioritisation of scarce vaccines in the future. Data came from statutory infectious disease notifications in educational institutions and from in-depth investigations by the district public health authorities. Using interrupted time series analyses, we measured the effect of mRNA-based vaccination of day-care staff on SARS-CoV-2 infections and transmission. Among 566 index cases from day-care centres, the mean number of secondary SARS-CoV-2 infections per index case dropped by −0.60 case per month after March 2021. The proportion of staff among all cases reported from daycares was around 60% in the pre-interruption phase and significantly decreased by 27 percentage points immediately in March 2021 and by further 6 percentage points each month in the post-interruption phase. Early vaccination of day-care staff reduced SARS-CoV-2 cases in the overall day-care setting and thus also protected unvaccinated children. This should inform future decisions on vaccination prioritisation.

Before vaccination against COVID-19 became widely available for all adults in Germany at the beginning of summer 2021, vaccines were scarce during the first half of 2021. In an effort to maximise the vaccination effect against morbidity and mortality in the population, the government prioritised certain population groups for vaccination. One such group was day-care staff, for whom prioritised vaccination started on 1 March 2021. About 25,000 day-care staff were vaccinated by the end of March 2021^1^; this was 70% of all day-care staff in Rhineland-Palatinate (https://impfdokumentation-rlp.de) [1]. By the end of June 2021, 84% of day-care staff had received both vaccination doses. As COVID-19 vaccination was not recommended for children, only few children with a high risk of severe COVID-19 due to pre-existing health conditions were vaccinated against COVID-19 during the study period. With less than one-fifth of people in daycares being staff and >80% being children, this led to a dilution of vaccination coverage to <20% in daycares overall (both children and staff).

Despite this low coverage, prioritising day-care staff may still have led to a reduction in transmission risk to unvaccinated individuals (i.e. indirect vaccination effect) by the following mechanisms: (i) reducing the risk of infection for vaccinated individuals and thus the contacts of unvaccinated individuals with infectious individuals and/or (ii) reducing the infectiousness of vaccinated individuals once they became infected. Previous studies provide evidence for indirect vaccination effects in the general population [2,3] and in households [4-6], but similar findings from the educational context or in other settings have not been published so far. Knowledge about indirect effects in daycares is of strategic importance when managing a pandemic or an outbreak as the number of people working in this setting is manageable, and day-care staff are easier to target with interventions than private households. It is the aim of this study to evaluate the direct and indirect vaccination effects on SARS-CoV-2 transmission in the day-care setting following vaccination prioritisation of day-care staff in early 2021.

The presented data were collected in the German federal state of Rhineland-Palatinate with about 4 million inhabitants, including 143,000 children attending daycares in 2021 [1]. During the study period, several dynamic non-pharmaceutical measures were in place to prevent the transmission of COVID-19 in daycares and in schools (Supplementary Table 1). Among these measures were face masks (mainly in schools), cohort/group separation, full or partial closures of institutions at the start of 2021, and a rapid antigen testing strategy starting from April 2021.

In the German public health system, the routine for COVID-19 control started with the statutory notification of an identified COVID-19 case, followed by an interview of this index case by qualified personnel at the district public health authority in charge. Subsequently, all contact people to the index case were traced, and quarantine of high-risk contacts was initiated, including initial PCR testing and an active follow-up for 14 days after the last contact with that index case. A high-risk contact was defined as a person who stayed face to face (<1.5 meters) with a COVID-19 case for 15 minutes or longer, or in the same room (i.e. irrespective of distance) for 30 minutes or longer [7].

By German law, all COVID-19 cases that have been identified by the district public health authorities were subsequently reported to the Rhineland-Palatinate public health authority, including information on age, sex, date of disease onset and date of reporting, symptom status, and whether a case attended or worked in an educational institution (i.e. schools and daycares). For this study, a case was defined as a person testing positive for SARS-CoV-2 in PCR irrespective of symptom status. In August 2020, the Rhineland-Palatinate public health authority initiated SARS-S (secondary attack rates in schools – surveillance), which collected additional data on the reported index cases in educational institutions and their respective contacts from the district authorities using a two-page data form [8]. Within SARS-S, a secondary case was defined as an individual who (i) was identified as a high-risk contact person to an index case in the educational setting by the responsible district authority, (ii) tested positive for SARS-CoV-2-RNA during the quarantine associated with that index case and (iii) was unlikely a co-primary case based on evidence on the assumed chain of infection that evolved during the competent district authority’s investigation. Contact people and secondary cases not attending the educational institution, for example, people living in the same household, were not reported via this data form.

The exposure of interest was vaccination of day-care staff, which was available on the federal state level (https://impfdokumentation-rlp.de). Due to the absence of individual-level vaccination data, we performed an interrupted time series analysis (ITSA) to assess the effect of vaccination on SARS-CoV-2 transmission. The concept of ITSA briefly is the estimation of an offset and a trend based on data from the pre-interruption period (October 2020 to February 2021) and the prediction of values for the post-interruption period (March 2021 to June 2021) based on the pre-interruption data. The predicted values based on the pre-interruption data are then compared to estimated values based on the post-interruption period. Significant differences support an association between the outcome of interest and the interruption point. Here we chose a linear model and monthly intervals with a Prais-Winsten estimation and set the interruption point to March 2021, when vaccines were made available to all persons working in daycares.

Two different outcomes were used to assess the effect of day-care staff vaccination in ITSA: (A) SARS-CoV-2 transmission in daycares overall and (B) the risk of infection for day-care staff relative to children. For the first outcome (A), data from the SARS-S study were used to calculate the mean number of secondary cases per index case with exact 95% confidence intervals for each month between October 2020 and June 2021. For the second outcome (B), routine notification data were used to calculate the proportion of reported COVID-19 cases in day-care staff among all cases reported from daycares with exact 95% confidence intervals for each month during the same period.

COVID-19 has been shown to vary with seasonality [9]. Vaccination in daycares started in March and thus coincided with spring, when we would have expected a potential natural decrease in cases due to warmer temperatures. However, such a seasonality-related decrease would likely have affected the overall population, rather than daycares alone. For this reason, we decided to control for seasonality in a sensitivity analysis, for which we considered schools as a suitable control group to provide evidence that the decrease of COVID-19 transmission in daycares was not caused by seasonality. We performed additional ITSAs with a control group consisting of index cases from schools, where vaccination had been offered neither to staff nor to children during the study period, with the exception of primary school teachers. Stata SE (version 16.1) with the ITSA module was used for data preparation and for conducting ITSAs. GraphPad Prism was used to visualise the results [10,11].

Between October 2020 and June 2021, the district public health authorities in Rhineland-Palatinate were notified about 144,000 cases of COVID-19. An estimated 94,000 of these cases were caused by the wildtype (mainly October 2020 to February 2021) and about 50,000 were caused by the alpha variant (mainly March to June 2021) of the SARS-CoV-2 virus.

Information on 566 index cases from daycares resulting in 712 secondary cases among day-care contacts was reported to SARS-S between October 2020 and June 2021 (Figure 1, panel A). On average, each index case was associated with 1.26 secondary cases. Nearly 70% of index cases did not lead to any secondary cases, about 10% were associated with exactly one secondary case, and the maximum number of secondary cases identified around one index case was 34. The highest mean numbers of secondary cases were reported during February and March 2021, the lowest number in June 2021. The ITSA shows that the mean number of secondary cases from the SARS‑S study changed significantly after vaccinations began for day-care staff in March 2021 (interruption point). Starting after March, there was a significant drop in the slope of −0.60 mean secondary cases per index case each month (95% CI –0.92, –0.27 (Supplementary Table 2)). The absence of a significant drop in the offset at the interruption point (0.34, 95% CI –1.02, 1.71) indicates a lag time between start of vaccination and the reduction in the number of secondary cases per index.

**Figure 1.**
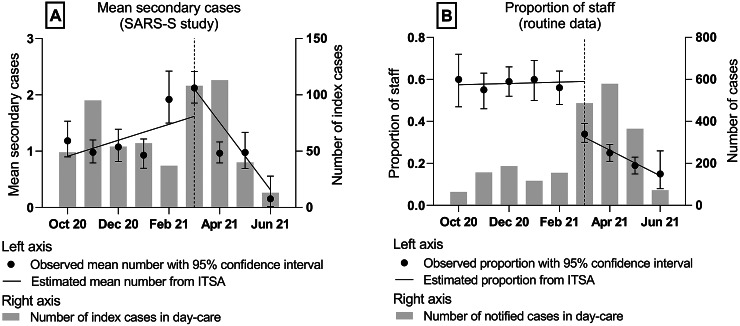
Mean secondary cases per index case (A) and proportion of identified cases from day-care staff among all identified day-care cases (B), before and after the start of SARS-CoV-2 vaccination of day-care staff in March (dotted line), Rhineland-Palatinate 2020–21.

During the same period, 2163 cases in daycares were reported via the German statutory notification system (Figure 1, panel B). Of these, 776 cases (36%) were day-care staff. There was an immediate strong decrease in the proportion of day-care staff from 59% to 32% at the interruption point in March 2021 (offset change −0.27, 95% CI –0.32, –0.21), followed by a linear decrease of 6 percentage points per additional month afterwards (slope change –0.06, 95% CI –0.08, –0.04 (Supplementary Table 2)). The figure shows that the modelled estimates align well with the observed data.

In controlled ITSAs, no significant changes were identified in the mean number of secondary cases (SARS-S (A)) or the proportion of staff among cases (routine surveillance (B)) in the school setting (control group), while results for the day-care setting were comparable with the main uncontrolled analysis (Supplementary Table 3).

The results of ITSA show that the initiation of mRNA-based vaccination of day-care staff was followed by a significant decrease in the mean number of secondary cases per index in the day-care setting (panel A). The first effects became visible in April 2021. This aligns with the expected timing of an effect, which would start from 2 weeks after the first dose and be highest from at least 1 week after the second dose [12]. This outcome measure includes both direct and indirect vaccination effects: before the start of the intervention, the average proportion of staff among all secondary cases in the SARS-S study was 53% (data not shown). Thus, keeping everything else constant, a 100% direct vaccination effect could have reduced the mean number of secondary cases by a maximum of 53%; that is, due to vaccination, none of the day-care staff became infected, and that infections would only occur among children. The observed and estimated decreases, however, were much higher (about 90%, derived from a drop in estimated mean secondary cases per index case from February 2021 (1.47) to June 2021 (0.15); Figure 1, panel A), so indirect vaccination effects on unvaccinated child contacts have most likely been involved.

As for the proportion of day-care staff among all identified cases from daycares (panel B), there was an immediate effect in March 2021, just after the start of vaccination. Although early vaccination effects could show from 2 weeks after receiving the first dose, the immediate drop seen in the data seems unlikely to be a pure effect of vaccination. An alternative explanation could be increased awareness of infection risk at times of increasing incidence of the disease plus the start of vaccination. If day-care staff behaved more cautiously during this time, also outside the day-care setting, this could have led to a lower number of SARS-CoV-2 cases in this group. The continuous decrease in the proportion of day-care staff among all identified cases from daycares from March 2021 onwards parallels the increase in the number of partly and fully vaccinated day-care staff and thus provides evidence for an increasing direct vaccination effect. Of note, this part of the analysis (panel B) was not designed to analyse indirect vaccination effects.

As the results presented are based on longitudinal aggregated data without individual-level information about vaccination status, a number of alternative explanations for the observed effects have to be taken into consideration.

Firstly, one alternative explanation could be changes in the background population incidence with increased reproduction numbers. However, the mean number of secondary cases per index case is largely independent of the background incidence, as only secondary cases to the specific index cases were considered. Also, one would expect the mean number of secondary cases to increase with increasing population incidence, which is the opposite to the presented findings. In addition, the absence of a significant change at the interruption point in the school index cases in sensitivity analysis from SARS-S makes this alternative explanation unlikely.

Secondly, different testing strategies in daycares could explain the lower number of secondary cases after vaccination introduction. Indeed, regular biweekly self-testing of day-care and school staff started in April 2021 with the ultimate goal of reducing the number of secondary cases based on an early identification of asymptomatic infectious cases. This means that before reaching this ultimate goal, the increased case detection should have led to an increase in the proportion of detected cases among staff as compared to children. However, the opposite was found in the analysis of routine data for both daycares and schools. Additionally, the observed proportion in April aligns well with the observed proportions in March and May (Figure 1, panel B), not indicating any visible effects of the new testing regime on the studied outcomes.

Thirdly, seasonal effects could have led to a reduction in transmission during spring, as compared to winter months. We could show that in the absence of any visible effects in the school setting, seasonal effects are unlikely to explain the strong effect that we observed in day-care centres. However, as non-pharmaceutical preventive measures and the age and population structure differed strongly between daycares and schools, results from sensitivity analysis have to be interpreted with caution as schools may not control adequately for potential confounding in all conceivable dimensions.

Fourthly, the presence of different variants of SARS-CoV-2 during the study period could explain part of the observed effects. During the study period, the pandemic in Germany was dominated by the wildtype and the alpha variant of concern. While cases were exclusively infected with the wildtype up until December 2020, about 90% of cases were caused by the more infectious alpha variant in April and May 2021 on average [13,14]. The even-more-infectious delta variant only played a minor role during the study period (about 3% of cases in May and 39% of cases at the end of the study period in June 2021). The surge of the more infectious alpha variant in the beginning of 2021 aligns well with the increased mean number of secondary cases in February and March 2021, but could not explain the strong decrease from March onwards.

This is the first study to provide evidence for a strong protective effect of staff vaccination on transmission of SARS-CoV-2 in the overall day-care setting. The magnitude of these findings suggests that this effect goes beyond direct protection of vaccinated individuals. Indeed, a previous study in a German day-care setting found that transmission from infected staff to both staff and children occurs twice as often as transmission from infected children to both staff and children [8], which provides a potential explanation of the strong effect of vaccination of staff only on the overall transmission in the day-care setting.

A meta-analysis involving studies from Israel, the Netherlands and Germany during the time of the alpha variant showed strong indirect vaccination effects on transmission. Unvaccinated individuals were three times less likely to become infected if the index case in the same household was vaccinated against COVID-19, than if the index case was unvaccinated [5]. The day-care setting is comparable to the household setting in several aspects: individuals eat together, use the same toilet, and neither children nor staff wear facemasks. While individuals are usually expected to stay home from daycares when experiencing disease symptoms, this provides only limited protection with regard to SARS-CoV-2, as individuals can become infectious several days before the onset of symptoms [15].

This study provides evidence that prioritising day-care staff for vaccination resulted in a substantially reduced number of SARS-CoV-2 cases in day-care staff and children. This shows that indirect effects of vaccination of a small proportion of people (<25% of people in day-care are staff) with a high number of unprotected contacts can largely mitigate disease transmission. This knowledge can help future decision-making in pandemics or with future variant-specific SARS-CoV-2 vaccines when one of the major aims is to keep day-care facilities open.

## Data Availability

Data and analytic code will be shared immediately following the publication of the article by the corresponding author upon reasonable request.

## Footnotes

1 Main vaccines used were mRNA-based, that is, Comirnaty® (BioNTech/Pfizer) and Spikevax® (Moderna).

## Supplementary material

For supplementary material accompanying this paper visit https://doi.org/10.1017/S0950268823000638. click here to view supplementary material
