# Supplementary material for: Direct and indirect vaccination effects on SARS-CoV-2 infection in day-care centres: Evaluating the policy for early vaccination of day-care staff in Germany, 2021
Source: Epidemiol Infect. 2023 May 4;151:e80. doi: 10.1017/S0950268823000638 (PMC10203534; doi:10.1017/S0950268823000638)
Supplement: Supplementary file 1 [file S0950268823000638sup001.docx]

Supplementary Table 1: Overview of COVID-19 non-pharmaceutical measures in day care and schools during the study period September 2020 to June 2021 in Rhineland-Palatinate, Germany

Supplementary Table 2: Interrupted time series analysis with outcome mean number of secondary cases (SARS-S, left) and proportion of staff among all day-care cases (routine surveillance, right)

| **Month** | **SARS-S study: mean secondary cases per index** | | | **Statutory notifications: proportion of staff among cases** | | |
| --- | --- | --- | --- | --- | --- | --- |
|  | **Beta coefficient** | **p-value** | **95% CI^#^** | **Beta coefficient** | **p-value** | **95% CI^#^** |
| Pre-Intervention phase (Oct-Feb) |  |  |  |  |  |  |
| offset | 0.91 | 0.003 | 0.47;1.35 | 0.57 | <0.001 | 0.55;0.60 |
| slope | 0.14 | 0.22 | -0.12;0.40 | 0.003 | 0.54 | -0.01;0.01 |
| Post-Intervention phase (Mar-Jun) |  |  |  |  |  |  |
| offset change | 0.34 | 0.55 | -1.02;1.71 | -0.27 | <0.001 | -0.32;-0.21 |
| Slope change | -0.74 | 0.004 | -1.12;-0.36 | -0.06 | <0.001 | -0.08;-0.04 |
| Post-Intervention slope | -0.60 | 0.005 | -0.92;-0.27 | -0.06 | <0.001 | -0.08;-0.04 |

# CI – confidence interval

Supplementary Table 3: Sensitivity interrupted time series analysis with control group schools* and outcome mean number of secondary cases (SARS-S, left) and proportion of staff among all day-care cases (routine surveillance, right)

| **Month** | **SARS-S study: mean secondary cases per index** | | | **Statutory notifications: proportion of staff among cases** | | |
| --- | --- | --- | --- | --- | --- | --- |
|  | **Beta coefficient** | **p-value** | **95% CI^#^** | **Beta coefficient** | **p-value** | **95% CI^#^** |
| Pre-Intervention phase (Oct-Feb) |  |  |  |  |  |  |
| Offset control | 0.29 | <0.001 | 0.22;0.35 | 0.23 | <0.001 | 0.19;0.28 |
| Slope control | -0.08 | <0.001 | -0.11;-0.05 | -0.03 | 0.002 | -0.05;-0.01 |
| Offset difference intervention | 0.65 | 0.02 | 0.15;1.15 | 0.35 | <0.001 | 0.29;0.41 |
| Slope difference intervention | 0.22 | 0.09 | -0.04;0.48 | 0.03 | 0.01 | 0.01;0.05 |
| Post-Intervention phase (Mar-Jun) |  |  |  |  |  |  |
| Offset change control | 0.22 | 0.02 | 0.04;0.41 | 0.01 | 0.69 | -0.04;0.06 |
| Slope change control | 0.12 | 0.08 | -0.01;0.25 | 0.02 | 0.15 | -0.01;0.04 |
| Offset change difference intervention | 0.07 | 0.86 | -0.78;0.92 | -0.25 | <0.001 | -0.33;-0.18 |
| Slope change difference intervention | -0.85 | 0.001 | -1.24;-0.45 | -0.08 | <0.001 | -0.11;-0.05 |
| Post-Intervention slope control | 0.03 | 0.51 | -0.09;0.16 | -0.01 | 0.06 | -0.03;0.00 |
| Post-Intervention slope intervention | -0.59 | <0.001 | -0.77;-0.41 | -0.06 | <0.001 | -0.08;-0.05 |

*control group for SARS-S study: secondary schools, control group for routine surveillance: all schools, # CI – confidence interval
